# Supplementary material for: Sub-micro porous thin polymer membranes for discriminating H2 and CO2
Source: Nat Commun. 2024 Jan 20;15:628. doi: 10.1038/s41467-024-45007-6 (PMC10799960; doi:10.1038/s41467-024-45007-6)
Supplement: Supplementary file 1 — Supplementary Information [file 41467_2024_45007_MOESM1_ESM.pdf]

Supplementary information

Sub-micro porous thin polymer membranes for discriminating H_2 and CO_2

Xueru Yan[1, 2], Tianqi Song[3], Min Li[1, 2], Zhi Wang[1, 2], Xinlei Liu [1, 2*]

[1] Chemical Engineering Research Center, School of Chemical Engineering and Technology, Tianjin University, Tianjin 300072, China. [2] Tianjin Key Laboratory of Membrane Science and Desalination Technology, Haihe Laboratory of Sustainable Chemical Transformations, State Key Laboratory of Chemical Engineering, Tianjin University, Tianjin 300072, China. [3] School of Computer Science and Technology, Xi'an Jiaotong University, Xi'an 710049, China

The author supervised this work: Xinlei Liu, xinlei_liu1@tju.edu.cn

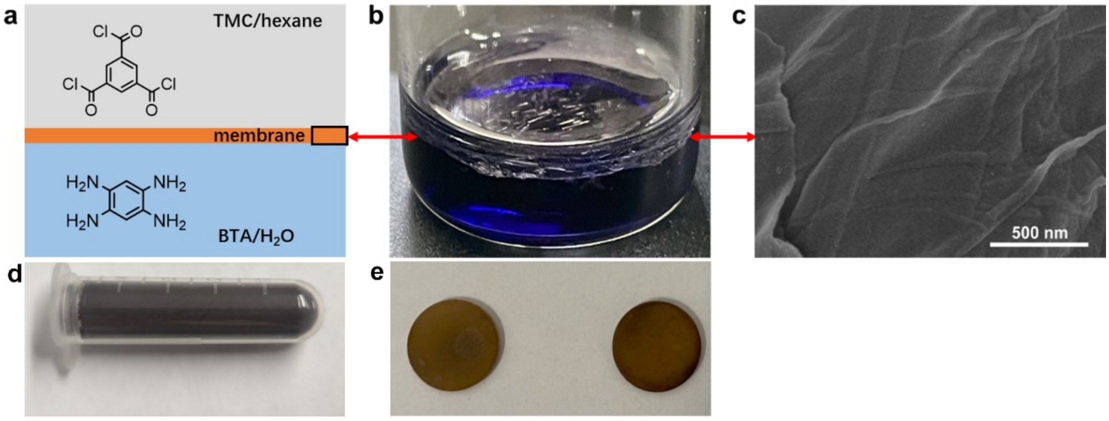

Supplementary Figure 1. **Synthesis and morphology of ALP films and membranes. a** Schematic process for synthesizing ALP film. **b** Photograph of an ALP film formed at the hexane-H_2O interface. **c** SEM image of an ALP film on a silicon wafer. **d** Photograph of BIALP200 (pH=1) powder. **e** Photograph of BIALP200 (pH=1) membranes (disk diameter = 18 mm).

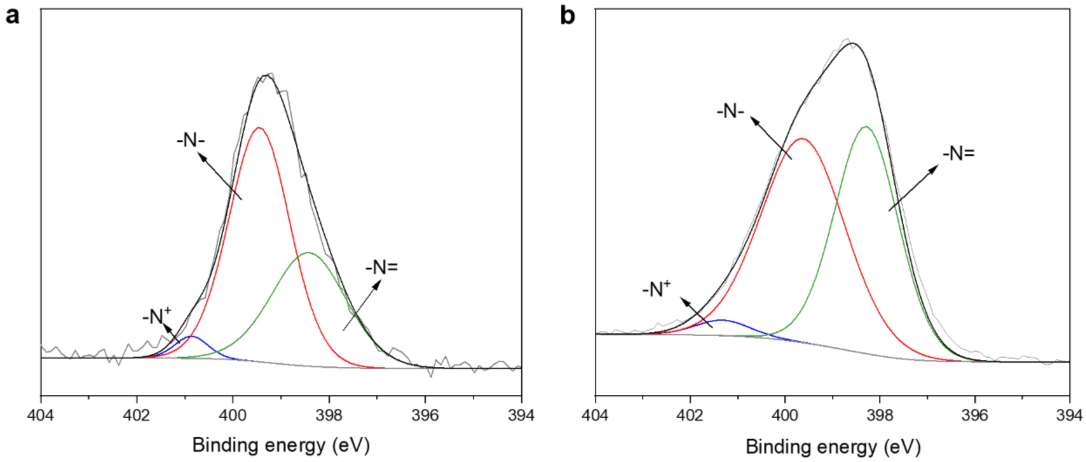

Supplementary Figure 2. **Narrow scans of *N1s* spectra. a** BIALP150 (pH=1) membrane. **b** BIALP300 (pH=1) membrane.

Supplementary Table 1. Effect of heating temperature on membrane composition. Binding energies and plausible species were determined from the deconvolution of *N1s* core level XPS spectra.

Species	Binding energy (eV)	Atomic ratio (%)			
		ALP	BIALP150	BIALP200	BIALP300
-N=	398.1-398.4	-	31.9	36.4	39.6
-N-	399.5-399.8	80.3	55.9	55.3	56.2
$-N^+$	400.7-401.2	19.7	12.2	8.3	4.2

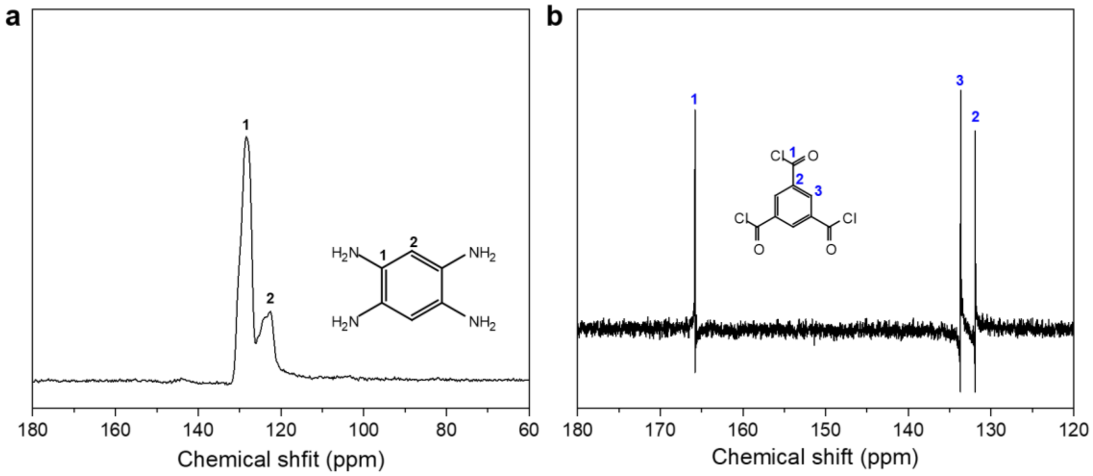

Supplementary Figure 3. **^{13}C NMR spectra of BTA and TMC monomers. a** ^{13}C solid-state NMR of the BTA. **b** ^{13}C liquid-state NMR of the TMC (DMSO-d6 was used as solvent).

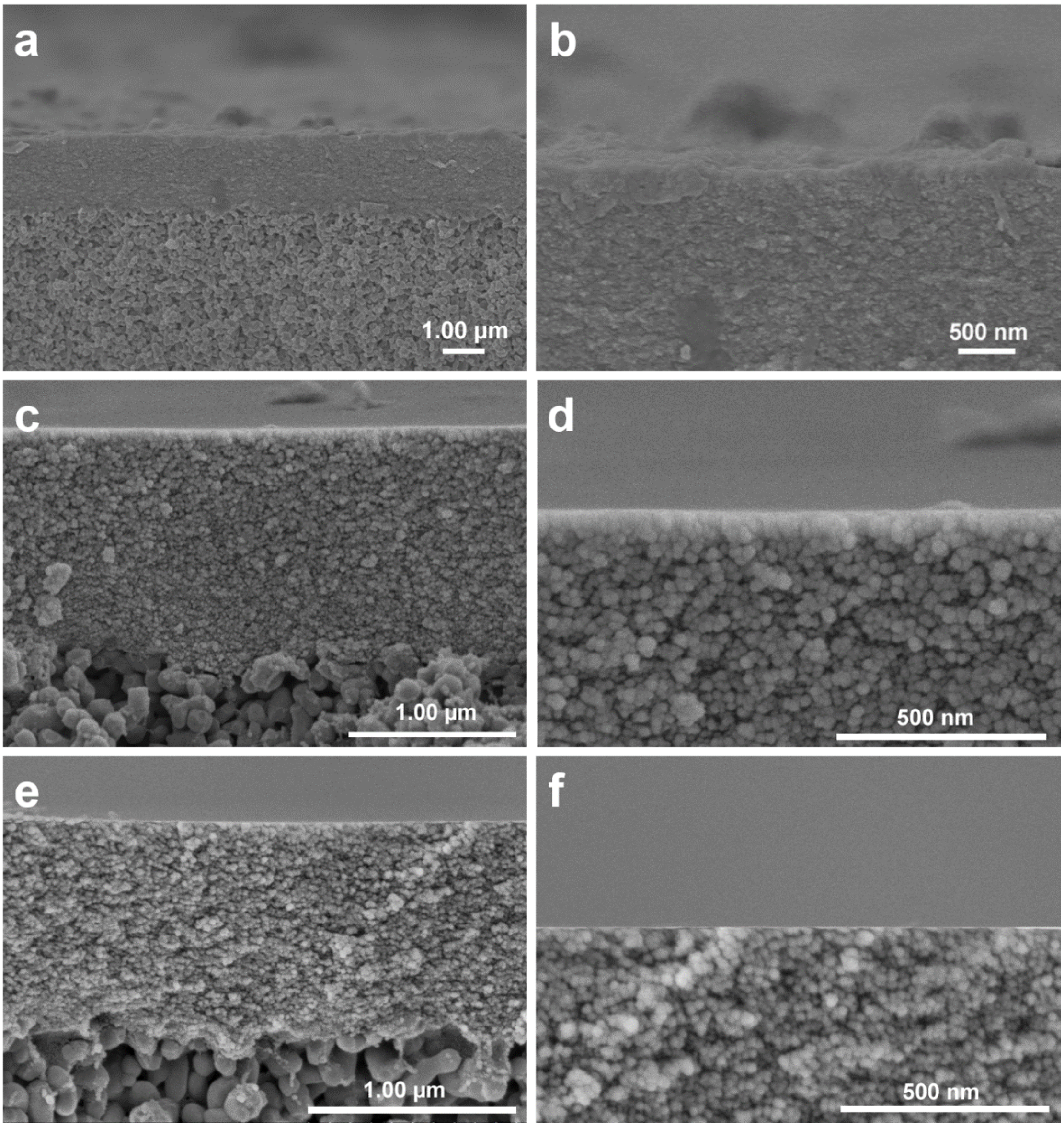

Supplementary Figure 4. **SEM cross-section images of BIALP membranes. a** and **b** BIALP150 (pH=1) membrane. **c** and **d** BIALP200 (pH=1) membrane. **e** and **f** BIALP300 (pH=1) membrane.

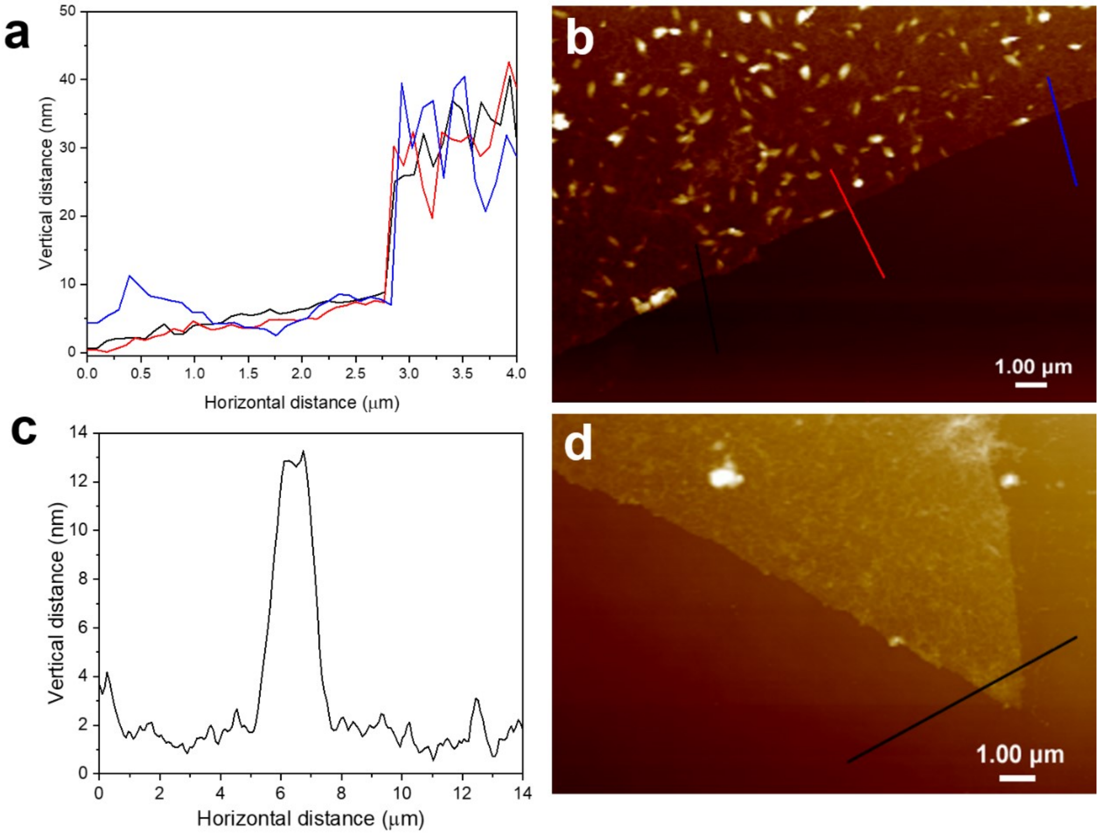

Supplementary Figure 5. **AFM characterizations of BIALP films on silicon wafers. a** and **b** Height profile and image of a BIALP150 (pH=1) film. **c** and **d** Height profile and image of a BIALP300 (pH=1) film.

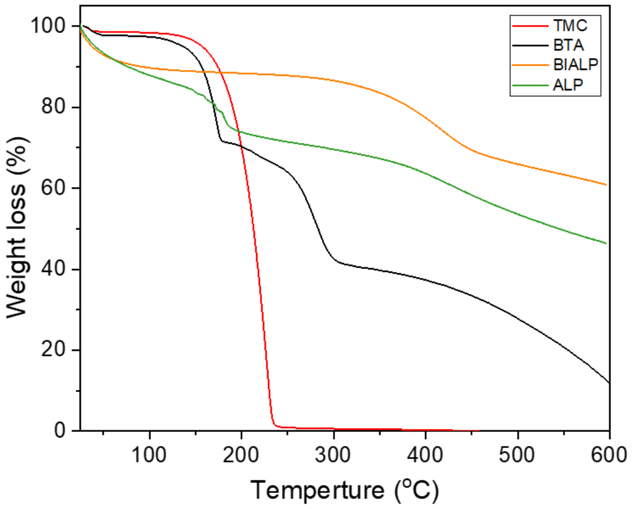

Supplementary Figure 6. **TGA curves of monomers (TMC and BTA), PA, and BIALP powders.** BIALP200 (pH=1) (orange line) and ALP (green line) powders were heated from room temperature to 600 °C at 5 °C/min in N_2. TMC (red line) and BTA (black line) monomers were heated from room temperature to 600 °C at 5 °C/min in air.

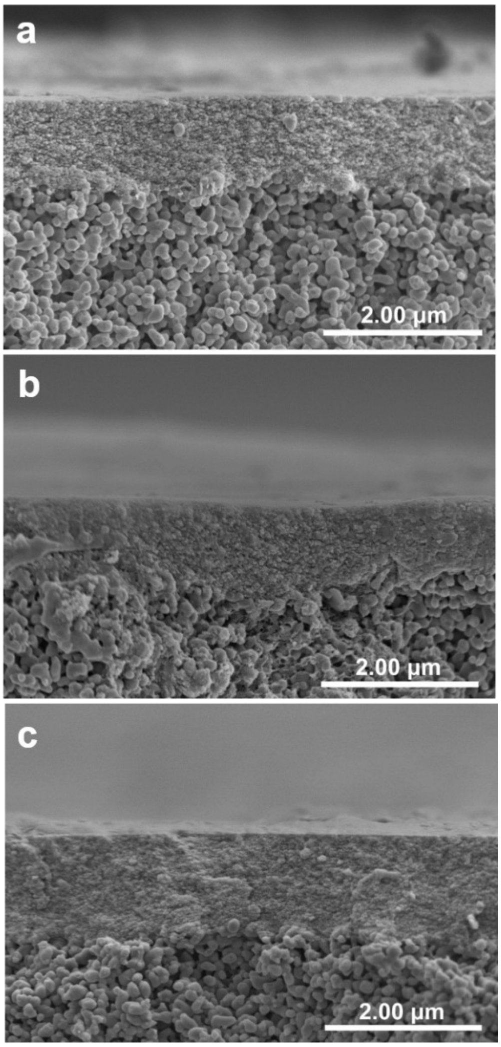

Supplementary Figure 7. **SEM cross-section images of BIALP membranes. a** BIALP200 (pH=1) membrane. **b** BIALP200 (pH=8) membrane. **c** BIALP200 (pH=13) membrane.

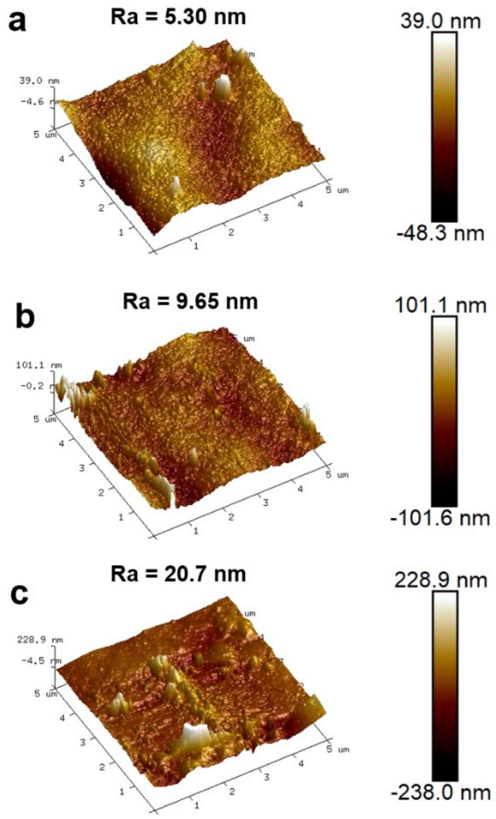

Supplementary Figure 8. **Three-dimensional morphologies and roughness of BIALP membranes. a** BIALP200 (pH=1) membrane. **b** BIALP200 (pH=8) membrane. **c** BIALP200 (pH=13) membrane.

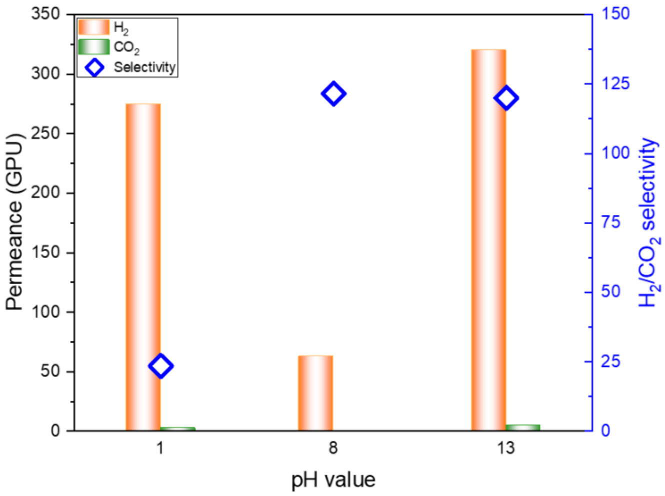

Supplementary Figure 9. **Effect of preadjusting pH value on BIALP200 membrane performances.** Testing conditions: H_2/CO_2 (1/1, mol/mol), 150 °C, 1 bar.

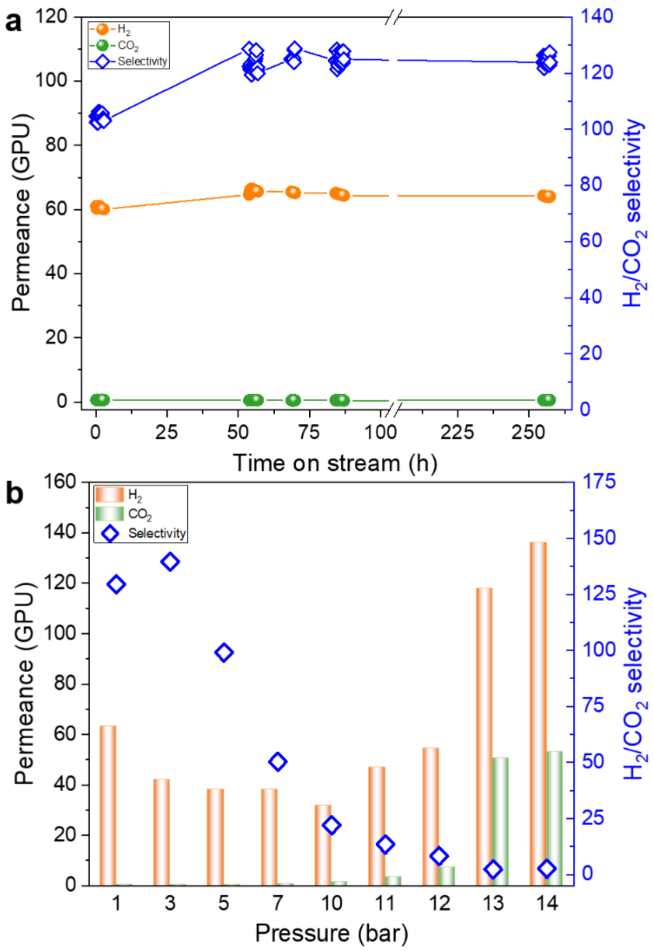

Supplementary Figure 10. **Stability and pressure-resistance test of BIALP200 (pH=8) membrane. a** Stability of BIALP200 (pH=8) membrane. **b** Pressure-resistance test of BIALP200 (pH=8) membrane. Testing conditions: H_2/CO_2 (1/1, mol/mol), 150 °C, 1 bar for **a** and pressure increased from 1 to 14 bar for **b**.

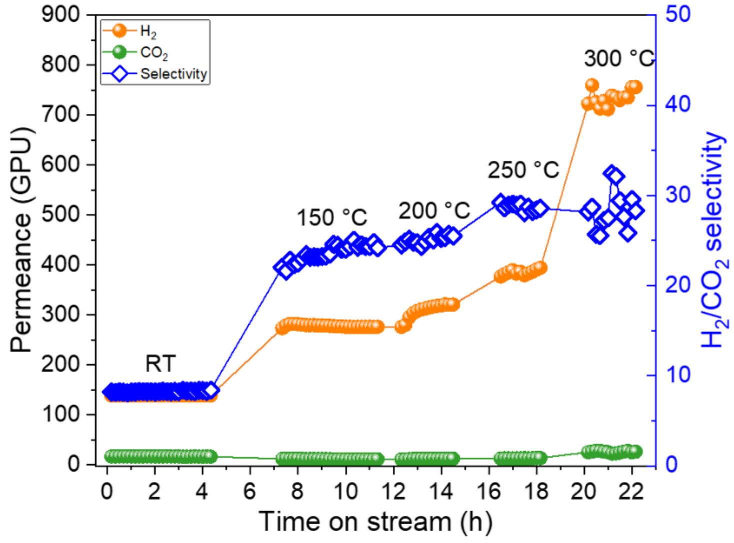

Supplementary Figure 11. **H_2/CO_2 separation by BIALP200 (pH=1) membranes.** Effects of testing temperatures on the separation performance. Testing conditions: H_2/CO_2 (1/1, mol/mol), 1 bar.

Supplementary Table 2. Synthetic parameters and separating performances of membranes in this work.

Membrane	Synthetic parameters		Test parameters		P_{H_2} (GPU)	α_{H_2/CO_2}
	pH	Heating temperature (°C)	Temperature (°C)	Pressure (bar)		
BIALP150	1	150	R.T.	1	65.4	29.1
			150	1	176	121
			200	1	405	44.3
			250	1	517	43.6
			300	1	795	25.1
			150	2	180	84.8
			150	3	187	80.2
			150	4	160	45.9
			150	5	140	43.4
			150	7	110	35.5
			150	10	41.9	35.8
			150	11	27.8	35.6
BIALP200	1	200	R.T.	1	145	9.63
			150	1	302	24.9
			200	1	321	25.3
			250	1	402	30.2
			300	1	750	33.6
BIALP300	1	300	R.T.	1	502	7.01
			150	1	504	40.2
			200	1	550	53.5
			250	1	597	58.1
			300	1	610	69.4
BIALP200	8	200	R.T.	1	46.5	59.9
			150	1	63	121
			150	3	45	147
			150	5	42	105
			150	7	44	61.6
			150	10	27	23.4
			150	11	51	12.0
			150	12	58	8.3
			150	13	120	6.7
			150	14	135	7.3
BIALP200	13	200	R.T.	1	302	32.1
			150	1	315	120

Feed stream: H_2/CO_2 (1/1, mol/mol) mixed gas.

R.T.: Room temperature.

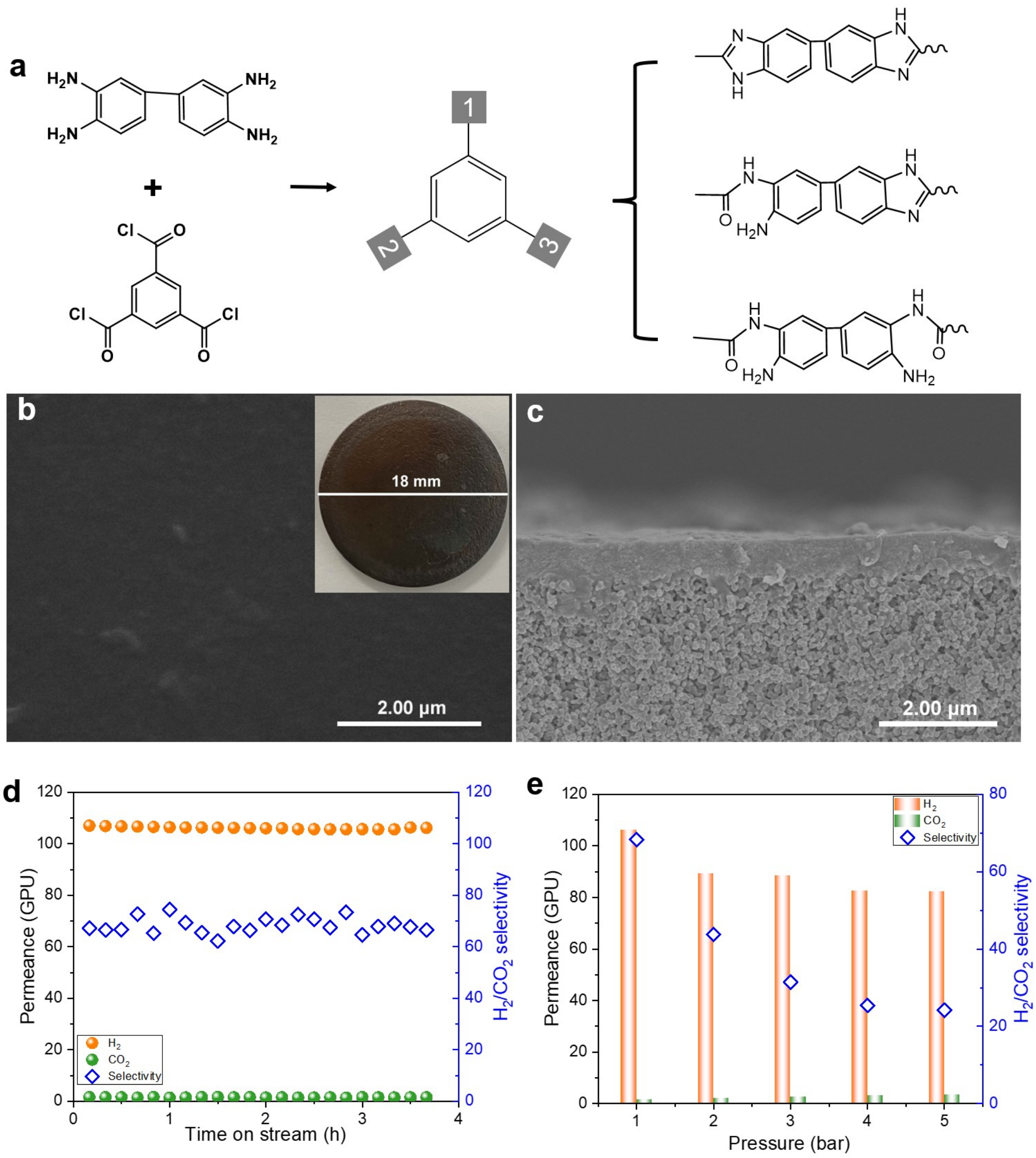

Supplementary Figure 12. **The versatility of the membrane synthetic approach via using the other amine monomer. a** Reaction scheme for forming a DAB-BIALP membrane. The three segments on the right part can be possibly connected to the numbered positions in the middle part. **b** SEM image (inset, digital photo) of top-surface DAB-BIALP150 (pH=1) membrane. **c** SEM image of cross-section DAB-BIALP150 (pH=1) membrane. d, Stability of a DAB-BIALP150 (pH=1) membrane. Feed conditions: equimolar H_2 and CO_2, 150 °C, 1 bar. **d** Effect of feed pressure on the performance of a DAB-BIALP150 (pH=1) membrane. Feed conditions: equimolar H_2 and CO_2, 150 °C.

To confirm the versatility of this fabrication method, 3,3'-diaminobenzidine (DAB) monomer was used instead of BTA using the same procedure as BIALP150 (pH=1) membranes. The membranes were noted as DAB-BIALP150 (pH=1) membranes. As shown in Supplementary Figure 12c, the membrane

had a similar ultra-thin thickness, roughly 40 nm.

The H_2/CO_2 separation performance of a DAB-BIALP150 (pH=1) membrane was tested at 150 °C, 1 bar (Supplementary Fig. 12d, e). Results confirmed that the H_2/CO_2 selectivity was around 70 corresponding to a H_2 permeance of 108 GPU. Furthermore, this membrane exhibited good pressure-resistance. Compared with the BIALP150 (pH=1) membranes, both the H_2 permeance and H_2/CO_2 selectivity were slightly lower. This is probably because the use of longer DAB monomer generated longer tightly-packed polymer chains, reducing the transport channels of H_2.

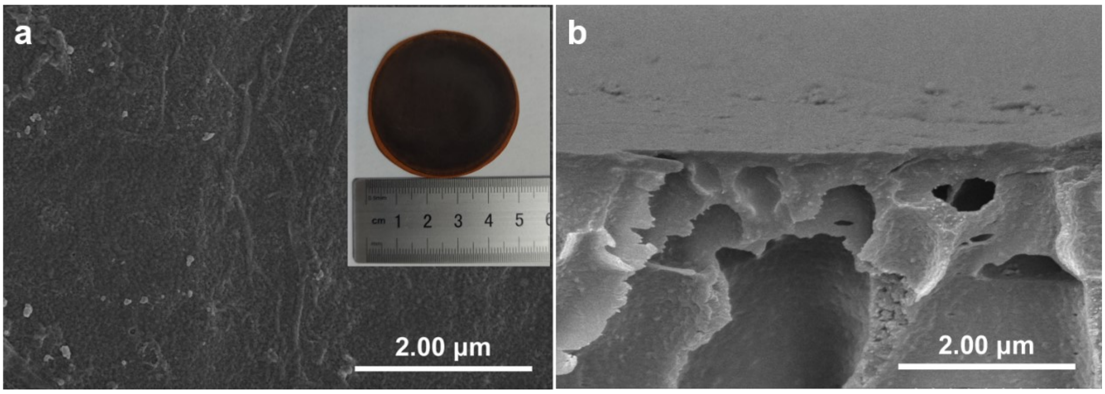

Supplementary Figure 13. **BIALP150 (pH=1) membranes formed on PAN substrates. a** SEM image (inset, digital photo) of surficial BIALP150 (pH=1) membrane on PAN substrate. **b** SEM image of cross-section BIALP150 (pH=1) membrane on PAN substrate.

Commercial polyacrylonitrile (PAN) membranes (Beijing Separate Equipment Co., Ltd, 50 KDa) were also selected as substrates. BIALP150 (pH=1) membranes were formed via the IP process mentioned in the main text followed by a thermal treatment at 150 °C. The SEM images and a digital photo of a BIALP membrane on PAN substrate are shown in Supplementary Fig. 13. The membrane showed a H_2/CO_2 selectivity of 62 corresponding to a H_2 permeance of 738 GPU at 150 °C and 1 bar.

Supplementary Table 3. Selected polymer membrane performances for H_2/CO_2 separation. The feed is H_2 and CO_2 mixture.

Membrane materials	Performance		Thickness (μm)	Operation conditions		Ref.
	P_{H_2}(GPU)	α_{H_2/CO_2}		Temperature (°C)	Pressure (bar)	
PBI	124	7.6	1*	150	3	1
	7.1	7.1	1*	35	7	2
	75	8.6	1*	180	7	2
	7	47	1*	250	3.4	3
	0.58	9.9	80	150	7	4
	20.3	35.6	1	250	6	5
	6.5	12.6	1	180	3	5
	20.3	35.6	1	250	6	5
	1.5	30	12	150	7.9	6
	0.72	18	12	100	11	7
	1.58	18	12	150	11	7
	3.17	17	12	300	11	7
	1.41	29	12	150	7	8
	12	34	1*	150	14	9
	182	22.2	0.300	150	1	10
	241	22.7	0.300	150	1	10
BILP-101x	24.2	39.5	0.400	150	1	11
	30.1	31.6	0.400	150	1	11
	257	38.5	0.0500	150	1	12
	127	56.3	0.0500	200	1	12
	322	33.4	0.0500	200	1	12
PA	350	59	0.1-0.3	140	-	13
COF	2163	26	0.0600	150	-	14
	669.1	24.2	0.450	25	1	15
	2566	22.6	0.01400	150	0.2	16
BIALP200(pH=13)	320	120	0.013	150	1	This work
BIALP150	174	121	0.0300	150	1	This work

1*: The membrane thickness was assumed to be 1 μm when it is not given.

Supplementary Table 4. Recently-reported other membrane performances for H_2/CO_2 separation. The feed is H_2 and CO_2 mixture.

Membrane		Operation conditions Temperature (°C)	Pressure (bar)	Thickness (μm)	PH_2 (GPU)	α_{H_2/CO_2}	Ref.
MOF	Co-gallate MOF	150	1.1	0.556	150	60	17
	$[Cu_2Br(IN)_2]n$	25	-	<0.01	527	294	18
	$Zn_2(bim)_4$	25	1	0.001	2700	291	19
	NH_2-$Zn_2(bim)_4$	R.T.	1	0.25	1095	1542	20
	$Zn_2(bim)_4$	R.T.	1	1*	1417	1158	21
	$Zn_2(bim)_4$	20	1	<0.01	2320	166	22
	$(Zn/Co)_2(bim)_4$	150	1	0.08	630	70	23
	ZIF-8	25	1	970	2654	17	24
	ZIF-95	25	2	0.4-0.5	571	184	25
	ZIF-95	200	1	20	493	41.6	26
	ZIF-95	R.T.	1	0.6	2291	32.2	27
	b-ZIF-L	240	1	25	1330	989	28
	ZIF-L	25	1	0.04	4033	321	29
	bMOF201	R.T.	1	62.7	1877	22.2	30
	NH_2-UiO-66	25	1	0.18	1230	41.3	31
	NH_2-UiO-66	20	1	4	1039	28.2	32
	UiO-67	R.T.	1	0.2	1809	8.9	33
	NH_2-MIL-53(Al)	80	1	15	686	23.7	34
	NH_2 -MIL-125(Ti)	30	1	0.5	129.3	22.8	35
	MAMS-1	40	1	0.04	880	225	36
	IRMOF-3	25	-	0.004	971	1473	37
	Ni-LAP	120	1	4.3	298	35	38
	KAUST-7	25	1	1-2	638	17.7	39
	KAUST-7	25	1	20	1537	27.3	40
GO	GO	25	-	0.05	10106	4	41
	q-rGO	25	-	1	783	3636	42
	GO	120	-	0.002	2000	47	43
	GO	20	-	0.009	344	3400	44
MXene	MXene	25	1	2	1113	167	45
	MXene	25	1	0.02	1200	20	46
	MXene	R.T.	1	0.22	70.6	30.3	47
	Pd-MXene	25	-	0.78	794	242	48
	MXene-ZIF-8	25	-	0.45	178	77	49
Zeolites	RUB-15	250	1	0.3	848	154	50
	RUB-15	225	2	0.15	160	30	51
	NaA	25	2	12.4	406	7.1	52
	ECNU-28	25	1	0.55	1272	61	53

1*: The membrane thickness was assumed to be 1 μm when it is not given.

R.T.: Room temperature.

References:

1. Sánchez-Laínez, J. *et al.* Influence of ZIF-8 particle size in the performance of polybenzimidazole mixed matrix membranes for pre-combustion CO_2 capture and its validation through interlaboratory test. *J. Membr. Sci.* **515**, 45–53 (2016).
2. Yang, T., Xiao, Y. & Chung, T.-S. Poly-/metal-benzimidazole nano-composite membranes for hydrogen purification. *Energy Environ. Sci.* **4**, 4171 (2011).
3. Berchtold, K. A., Singh, R. P., Young, J. S. & Dudeck, K. W. Polybenzimidazole composite membranes for high temperature synthesis gas separations. *J. Membr. Sci.* **415–416**, 265–270 (2012).
4. Naderi, A., Asadi Tashvigh, A., Chung, T. S., Weber, M. & Maletzko, C. Molecular design of double crosslinked sulfonated polyphenylsulfone /polybenzimidazole blend membranes for an efficient hydrogen purification. *J. Membr. Sci.* **563**, 726–733 (2018).
5. Sánchez-Laínez, J., Zornoza, B., Téllez, C. & Coronas, J. Asymmetric polybenzimidazole membranes with thin selective skin layer containing ZIF-8 for H_2/CO_2 separation at pre-combustion capture conditions. *J. Membr. Sci.* **563**, 427–434 (2018).
6. Hu, L., Bui, V. T., Huang, L., Singh, R. P. & Lin, H. Facilely cross-linking polybenzimidazole with polycarboxylic acids to improve H_2/CO_2 separation performance. *ACS Appl. Mater. Interfaces* **13**, 12521–12530 (2021).
7. Zhu, L., Swihart, M. T. & Lin, H. Tightening polybenzimidazole (PBI) nanostructure via chemical cross-linking for membrane H_2/CO_2 separation. *J. Mater. Chem. A* **5**, 19914–19923 (2017).
8. Hu, L. *et al.* Supramolecular assemblies of polybenzimidazole and aromatic polycarboxylic acids with superior mechanical and H_2/CO_2 separation properties. *J. Mater. Chem. A* **10**, 10872–10879 (2022).
9. Zhu, L., Swihart, M. T. & Lin, H. Unprecedented size-sieving ability in polybenzimidazole doped with polyprotic acids for membrane H_2/CO_2 separation. *Energy Environ. Sci.* **11**, 94–100 (2018).
10. Shan, M. *et al.* Novel high performance poly(*p*-phenylene benzobisimidazole) (PBDI) membranes fabricated by interfacial polymerization for H_2 separation. *J. Mater. Chem. A* **7**, 8929–8937 (2019).
11. Shan, M. *et al.* Facile manufacture of porous organic framework membranes for precombustion CO_2 capture. *Sci. Adv.* **4**, eaau1698 (2018).
12. Gao, A. *et al.* Designed channels in thin benzimidazole-linked polymer membranes for hot H_2 purification. *J. Membr. Sci.* **668**, 121293 (2023).
13. Ali, Z. *et al.* Ultra-selective defect-free interfacially polymerized molecular sieve thin-film composite membranes for H_2 purification. *J. Mater. Chem. A* **6**, 30–35 (2018).
14. Ying, Y., Peh, S. B., Yang, H., Yang, Z. & Zhao, D. Ultrathin Covalent Organic Framework Membranes via a Multi‐Interfacial Engineering Strategy for Gas Separation. *Adv. Mater.* **34(25)**, 2104946 (2022).
15. Fan, H. *et al.* Covalent organic framework–covalent organic framework bilayer membranes for highly selective gas separation. *J. Am. Chem. Soc.* **140**, 10094-10098 (2018).
16. Ying, Y. *et al.* Ultrathin two-dimensional membranes assembled by ionic covalent organic nanosheets with reduced apertures for gas separation. *J. Am. Chem. Soc.* **142**,

4472–4480 (2020).
17. Liu, Y. *et al.* Balancing the crystallinity and film formation of metal–organic framework membranes through in situ modulation for efficient gas separation. *Angew. Chem. Int. Ed.* **62**, e202309095 (2023).
18. Song, S. *et al.* Tuning the stacking modes of ultrathin two‐dimensional metal–organic framework nanosheet membranes for highly efficient hydrogen separation. *Angew. Chem. Int. Ed.* **62**, e202312995 (2023).
19. Peng, Y. *et al.* Metal-organic framework nanosheets as building blocks for molecular sieving membranes. *Science* **346**, 1356–1359 (2014).
20. Shu, L., Peng, Y., Song, H., Zhu, C. & Yang, W. Modular customization and regulation of metal–organic frameworks for efficient membrane separations. *Angew. Chem. Int. Ed.* e202315057 (2023) doi:10.1002/anie.202315057.
21. Song, H. *et al.* Structure regulation of MOF nanosheet membrane for accurate H_2/CO_2 separation. *Angew. Chem. Int. Ed.* **62**, e202218472 (2023).
22. Peng, Y., Li, Y., Ban, Y. & Yang, W. Two‐dimensional metal–organic framework nanosheets for membrane‐based gas separation. *Angew. Chem. Int. Ed.* **56**, 9757–9761 (2017).
23. Ma, C., Gao, G., Liu, H., Liu, Y. & Zhang, X. Fabrication of 2D bimetallic metal-organic framework ultrathin membranes by vapor phase transformation of hydroxy double salts. *J. Membr. Sci.* **644**, 120167 (2022).
24. Gao, Z. *et al.* Free-standing metal–organic framework membranes made by solvent-free space-confined conversion for efficient H_2/CO_2 separation. *ACS Appl. Mater. Interfaces* **15**, 19241–19249 (2023).
25. Deng, A. *et al.* Elimination of grain boundary defects in zeolitic imidazolate framework ZIF‐95 membrane via solvent‐free secondary growth. *Angew. Chem. Int. Ed.* **60**, 25463–25467 (2021).
26. Ma, X., Li, Y. & Huang, A. Synthesis of nano-sheets seeds for secondary growth of highly hydrogen permselective ZIF-95 membranes. *J. Membr. Sci.* **597**, 117629 (2020).
27. Ma, X. *et al.* Anisotropic gas separation in oriented ZIF‐95 membranes prepared by vapor‐assisted in‐plane epitaxial growth. *Angew. Chem. Int. Ed.* **59**, 20858–20862 (2020).
28. Zhao, M. *et al.* Heat-driven molecule gatekeepers in MOF membrane for record-high H_2 selectivity. *Sci. Adv.* **9**, eadg2229 (2023).
29. Yang, K. *et al.* ZIF-L membrane with a membrane-interlocked-support composite architecture for H_2/CO_2 separation. *Sci. Bull.* **66**, 1869–1876 (2021).
30. Schulte, Z. M. *et al.* H_2/CO_2 separations in multicomponent metal-adeninate MOFs with multiple chemically distinct pore environments. *Chem. Sci.* **11**, 12807–12815 (2020).
31. Sun, Y. *et al.* Fabrication of highly oriented ultrathin zirconium metal‐organic framework membrane from nanosheets towards unprecedented gas separation. *Angew. Chem. Int. Ed.* **62**, e202216697 (2023).
32. Sun, Y., Song, C., Guo, X. & Liu, Y. Concurrent manipulation of out-of-plane and regional in-plane orientations of NH_2-UiO-66 membranes with significantly reduced anisotropic grain boundary and superior H_2/CO_2 separation performance. *ACS Appl.*

Mater. Interfaces **12**, 4494–4500 (2020).

33. Knebel, A. *et al.* Azobenzene guest molecules as light-switchable CO_2 valves in an ultrathin UiO-67 Membrane. *Chem. Mater.* **29**, 3111–3117 (2017).
34. Zhang, F. *et al.* Hydrogen selective NH_2 - MIL - 53(Al) MOF membranes with high permeability. *Adv. Funct. Mater.* **22**, 3583–3590 (2012).
35. Sun, Y. *et al.* In - plane epitaxial growth of highly *c* - oriented NH_2 - MIL - 125(Ti) membranes with superior H_2/CO_2 selectivity. *Angew. Chem. Int. Ed.* **57**, 16088–16093 (2018).
36. Wang, X. *et al.* Reversed thermo-switchable molecular sieving membranes composed of two-dimensional metal-organic nanosheets for gas separation. *Nat. Commun.* **8**, 14460 (2017).
37. Zhang, X., Wang, N., Li, H., Wang, Z. & Wang, H. IRMOF-3 nanosheet-filled glass fiber membranes for efficient separation of hydrogen and carbon dioxide. *Sep. Purif. Technol.* **318**, 123908 (2023).
38. Yan, T. *et al.* Facile synthesis of ultra-microporous pillar-layered metal–organic framework membranes for highly H_2-selective separation. *ACS Appl. Mater. Interfaces* **15**, 20571–20582 (2023).
39. Lv, J. *et al.* In-situ synthesis of KAUST-7 membranes from fluorinated molecular building block for H_2/CO_2 separation. *J. Membr. Sci.* **658**, 120585 (2022).
40. Lv, J. *et al.* Inorganic pillar center-facilitated counter diffusion synthesis for highly H_2 perm-selective KAUST-7 membranes. *ACS Appl. Mater. Interfaces* **14**, 4297–4306 (2022).
41. Yang, H. *et al.* Manipulating gas transport channels in graphene oxide membrane with swift heavy ion irradiation. *Sep. Purif. Technol.* **320**, 124136 (2023).
42. Zhang, X. *et al.* Precisely tailored graphene oxide membranes on glass fiber supports for efficient hydrogen separation. *J. Membr. Sci.* **675**, 121529 (2023).
43. Chi, C. *et al.* Facile preparation of graphene oxide membranes for gas separation. *Chem. Mater.* **28**, 2921–2927 (2016).
44. Li, H. *et al.* Ultrathin, molecular-sieving graphene oxide membranes for selective hydrogen separation. *Science* **342**, 95–98 (2013).
45. Ding, L. *et al.* MXene molecular sieving membranes for highly efficient gas separation. *Nat. Commun.* **9**, 155 (2018).
46. Shen, J. *et al.* 2D MXene nanofilms with tunable gas transport channels. *Adv. Funct. Mater.* **28**, 1801511 (2018).
47. Qu, K. *et al.* Self-crosslinked MXene hollow fiber membranes for H_2/CO_2 separation. *J. Membr. Sci.* **638**, 119669 (2021).
48. Wang, Q. *et al.* Palladium-intercalated MXene membrane for efficient separation of H_2/CO_2: Combined experimental and modeling work. *J. Membr. Sci.* **653**, 120533 (2022).
49. Hong, X. *et al.* Fast fabrication of freestanding MXene-ZIF-8 dual-layered membranes for H_2/CO_2 separation. *J. Membr. Sci.* **642**, 119982 (2022).
50. Dakhchoune, M. *et al.* Gas-sieving zeolitic membranes fabricated by condensation of precursor nanosheets. *Nat. Mater.* **20**, 362–369 (2021).
51. Dakhchoune, M., Duan, X., Villalobos, L. F., Avalos, C. E. & Agrawal, K. V. Hydrogen-sieving zeolitic films by coating zeolite nanosheets on porous polymeric support. *J.*

Membr. Sci. **672**, 121454 (2023).

52. Wei, X. L. *et al.* Effects of bubbles on the structure and performance of zeolite membranes. *J. Eur. Ceram. Soc.* **40**, 1709–1716 (2020).

53. Wang, J. *et al*. Layered zeolite for assembly of two-dimensional separation membranes for hydrogen purification. *Angew. Chem. Int. Ed.* **62**, e202304734 (2023).
