# Supplementary material for: Sub-micro porous thin polymer membranes for discriminating H2 and CO2
Source: Nat Commun. 2024 Jan 20;15:628. doi: 10.1038/s41467-024-45007-6 (PMC10799960; doi:10.1038/s41467-024-45007-6)
Supplement: Supplementary file 3 — Description of Additional Supplementary Files [file 41467_2024_45007_MOESM3_ESM.pdf]

Description of Additional Supplementary Files

File Name: Supplementary Movie 1
Description: Mechanical strength of ALP film.
